# Meta-Analysis of miR-146a Polymorphisms Association with Coronary Artery Diseases and Ischemic Stroke

**DOI:** 10.3390/ijms160714305

**Published:** 2015-06-24

**Authors:** Mei-Hua Bao, Yan Xiao, Qing-Song Zhang, Huai-Qing Luo, Ji Luo, Juan Zhao, Guang-Yi Li, Jie Zeng, Jian-Ming Li

**Affiliations:** 1 Department of Anatomy, Histology and Embryology, Changsha Medical University, Changsha 410219, China; E-Mails: zhangqingsong@whut.edu.cn (Q.-S.Z.); luohuaiqing@163.com (H.-Q.L.); luoji927@163.com (J.L.); zhaojuannanfang2@163.com (J.Z.); liguangyi1977@163.com (G.-Y.L.); zengjie84117@163.com (J.Z.); 2 Qingdao Science & Standard Chemicals Analysing and Testing Co., Ltd., Qingdao 266000, China; E-Mail: y_xiao@sscta.cn

**Keywords:** miR-146a polymorphism, rs2910164, coronary artery disease, ischemic stroke, meta-analysis

## Abstract

Coronary artery disease (CAD) and ischemic stroke (IS) are manifestations of atherosclerosis, with a high death rate. miR-146a is a microRNA that participates in the progress of CAD and IS. A single nucleotide polymorphism (SNP) in the precursor of miR-146a, rs2910164, was found to be associated with the risks of CAD and IS. However, the results were inconsistent and inconclusive. A meta-analysis was performed to assess the relationship of rs2910164 and CAD as well as IS susceptibility. The database Pubmed, Embase, Cochrane Central Register of Controlled Trials (CENTRAL), Chinese National Knowledge Infrastructure (CNKI), and Chinese Biomedical Literature Database (CBM) were searched for related studies. Crude odds ratios with 95% confidence intervals were used to investigate the strength of the association by random- or fixed-effect model. A total of eight studies, with 3138 cases and 3097 controls were identified for the meta-analysis. The results shows that rs2910164 is associated with the risk of CAD significantly in allelic model (OR = 0.86), homozygous model (OR = 0.70), heterozygous model (OR = 0.80) and dominant model (OR = 0.76). The subjects carrying the GG genotype, GG + GC genotype or G allele are at lower risks of CAD. For the susceptibility of IS, there are no significant associations between rs2910164 and total studies. However, in subgroup analysis by sample size and ethnicity, the GG, GG + GC and G allele of rs2910164 are found to be associated with higher risks of IS in large sample size group and in Koreans, under homozygous and dominant models. In conclusion, the current meta-analysis suggests lower risks of CAD for GG, GG + GC genotype and G allele of rs2910164, while rs2910164 is not associated with the risk of IS. Thus rs2910164 might be recommended as a predictor for susceptibility of CAD, but not IS.

## 1. Introduction

Cardiovascular diseases are the major cause of death and disability worldwide. According to the World Health Organization report in 2011, more than 17 million people died of cardiovascular diseases. Coronary artery disease (CAD) is characterized by occlusive epicardial coronary artery stenosis. Ischemic stroke (IS) is a major kind of stroke, which causes a high death rate and adult disability in the world. According to the report of Liu, the annual stroke mortality rate was 120–180 per 100,000 in China [1]. In the United States, stroke ranks as the third leading cause of death [2]. IS has exceeded heart diseases to become the most frequent cause of death. IS and CAD are principal clinical manifestations of atherosclerosis, and have caused a huge burden for society. Previous studies demonstrated that single nucleotide polymorphisms (SNPs) were associated with the risks of CAD and IS [3,4].

MicroRNAs are small, non-coding RNAs which regulate the gene expression in post-transcriptional levels. The regulation effects of microRNAs are obtained by binding to the 3ʹ-UTR of target mRNAs, and lead to degradation or translation repression of target genes. Previous reports predicted that 1/3 of human genes were regulated by microRNAs [5]. Thus, the microRNAs interfere with many physiologic and pathological processes. The changes in the sequences of microRNAs, such as single nucleotide polymorphisms (SNPs), may result in diseases.

miR-146a is a microRNA located at the human chromosome 5q33. Previous studies have demonstrated that miR-146a participates in inflammatory processes, thus interferes with the pathology of cardiovascular diseases [6]. A SNP has been found to exist at the precursor miRNA-146a, which mutates G:U to C:U and results in a low production of mature miR-146a [7]. Studies have reported the relationship between miR-146a polymorphism and susceptibility of coronary artery disease and ischemic stroke. However, the results are somehow inconsistent. For example, some studies demonstrated that miR-146a polymorphism was associated with the risk of CAD and IS [8,9,10,11,12], while others thought it was not [13,14].

Thus it is necessary to make a precise and comprehensive estimation of the association between miR-146a and the risks of CAD and IS. In the present study, eight studies, including 3138 cases and 3097 controls, were included in the meta-analysis.

## 2. Results

### 2.1. Characteristics of Eligible Studies

**Figure 1:**
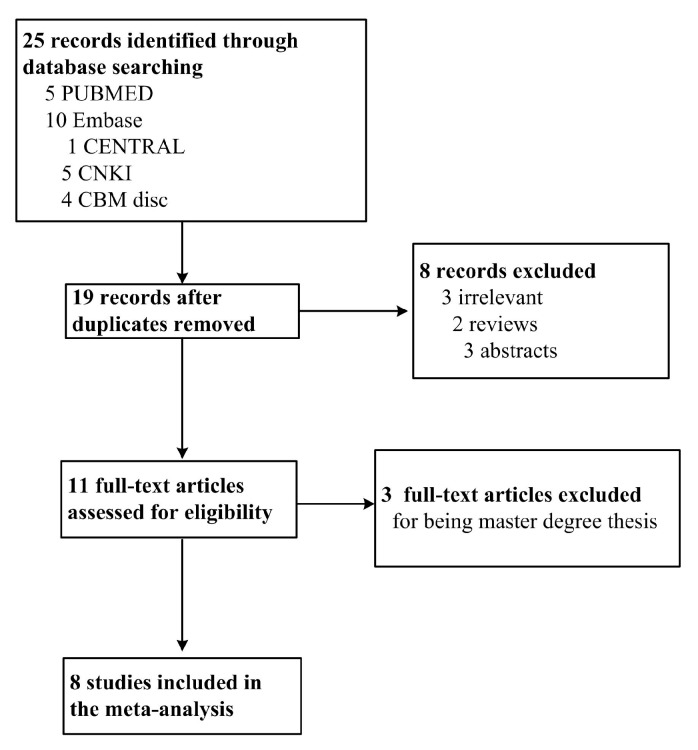
PRISMA flow chart of studies inclusion and exclusion.

A total of 19 studies were obtained from the literature search after duplicates were removed. Among them, three studies were excluded for irrelevance, two for being reviews, three for being master degree theses and three were only abstracts. Finally, eight studies meeting the criteria were preserved, which included 3138 cases and 3097 controls. The Preferred reporting items for systematic reviews and meta-analyses (PRISMA) flow chart is shown in Figure 1 and the information for the selected studies was presented in Table 1.

### 2.2. Results of Meta-Analysis

**Figure 2:**
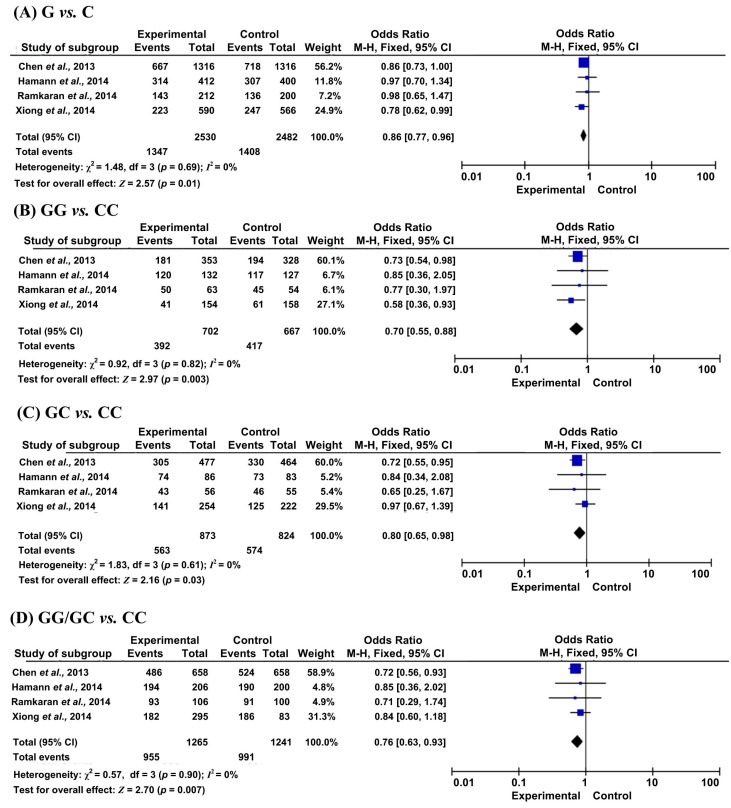
Forest plots of odds ratios for the association between microRNA-146a rs2910164 and the risk of CAD. (**A**) G *vs*. C; (**B**) GG *vs*. CC; (**C**) GC *vs*. CC; (**D**) GG/GC *vs*. CC.

**Figure 3:**
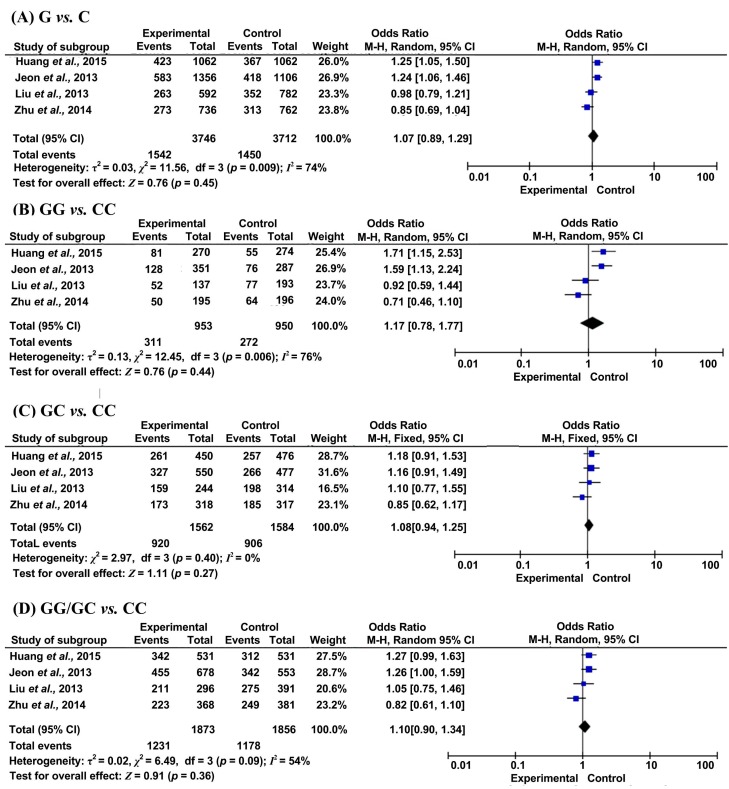
Forest plots of odds ratios for the association between microRNA-146a rs2910164 and risk of IS. (**A**) G *vs*. C; (**B**) GG *vs*. CC; (**C**) GC *vs*. CC; (**D**) GG/GC *vs*. CC.

The results of meta-analysis for the association between miR-146a (rs2910164) and CAD, IS risks were shown in Table 2 and Figure 2 and Figure 3.

For CAD, significantly decreased risks were found to be associated with rs2910164 under all genetic models, allelic model (OR = 0.86, 95% CI = 0.77–0.96, *p* = 0.01), homozygous model (OR = 0.70, 95% CI = 0.55–0.88, *p* = 0.003), heterozygous model (OR = 0.80, 95% CI = 0.65–0.98, *p* = 0.03) and dominant model (OR = 0.76, 95% CI = 0.63–0.93, *p* = 0.007). When we conducted a subgroup analysis by sample size and ethnicity, the same significant associations were observed in the large sample size group and the Chinese group. However, in the small sample size group, a significant decrease in CAD risks was only found in the homozygous model.

On the other hand, no significant association was found between rs2910164 and IS susceptibility in the analysis as a whole. However, subgroup analysis by sample size indicated a significant association between rs2910164 and IS susceptibility in large sample size groups and in Koreans, under homozygous and dominant models (Table 2). No other evident associations between rs2910164 and risk of IS were observed among subgroup analysis by Trial of Org 10,172 in Acute Stroke Treatment (TOAST), gender, smoking, hypertension, diabetes mellitus and hyperlipidemia in the dominant model (Table 3).

**Table 1:** Characteristics of eligible studies included in the meta-analysis.

Author	Year	Country	Ethnicity	Disease	Genotyping Methods	Sex Ratio (Male:Female) (Case/Control)	Age (Case/Control)	Quality Score	Sample Size (Case/Control)	GG (Case/Control)	GC (Case/Control)	CC (Case/Control)	HWE of Control
Chen *et al.*	2013	China	Asian	CAD	Taqman	1:1.79/1:1.184	64.57 ± 10.33/ 64.08 ± 11.21	14	658/658	181/194	305/330	172/134	0.769
Hamann *et al.*	2014	German	Caucasian	CAD	HRM	Unavailable	Unavailable	12	206/200	120/117	74/73	12/10	0.748
Ramkaran *et al.*	2014	South Africa	Indian	CAD	PCR-RFLP	1:0/1:0	37.6 ± 0.40/ 37.5 ± 0.44	12	106/100	50/45	43/46	13/9	0.569
Xiong *et al.*	2014	Chian	Asian	CAD	PCR-RFLP	1:0.661/1:0.601	65.13 ± 11.86/ 62.59 ± 12.89	11	295/283	41/61	141/125	113/97	0.086
Huang *et al.*	2015	China	Asian	IS	Taqman	1:0.616/1:0.616	61.0 (54, 68)/ 63.0 (54, 70)	14	531/531	81/55	261/257	189/219	0.106
Jeon *et al.*	2013	Korea	Korean	IS	Taqman	1:0.441/1:0.496	64.16 ± 11.90/ 63.14 ± 10.19	15	678/553	128/76	327/266	223/211	0.589
Liu *et al.*	2013	China	Asian	IS	PCR-RFLP	1:0.608/1:0.581	67.52 ± 10.29/ 66.34 ± 11.07	12	296/391	52/77	159/198	85/116	0.65
Zhu *et al.*	2014	China	Asian	IS	PCR-LDR	1:0.688/1:0.685	61.62 ± 0.986/ 62.05 ± 0.982	12	368/381	50/64	173/185	145/132	0.952

HWE: Hardy-Weinberg equilibrium; CAD: Coronary Artery Disease; HRM: High-resolution melting; PCR-RFLP: polymerase chain reaction-restriction fragment length polymorphism; IS: ischemic stroke.

**Table 2:** Pooled ORs and 95% CIs of the association between miR-146a (rs2910164) and coronary artery disease (CAD) and ischemic stroke (IS).

Genetic Model	Overall or Subgroup	CAD				IS			
		*n*	OR (95% CI)	*p*-Value	*I*^2^ (%)	*n*	OR (95% CI)	*p*-Value	*I*^2^ (%)
G *vs.* C	Overall	5	0.86 (0.77, 0.96)	0.01	0%	4	1.07 (0.89, 1.29)	0.45	74%
	Large size	2	0.86 (0.73, 1.00)	0.05	NA	2	1.25 (1.11, 1.41)	0.0003	0%
	Small size	3	0.87 (0.73, 1.03)	0.10	0%	2	0.91 (0.78, 1.05)	0.20	0%
	Chinese	2	0.83 (0.73, 0.95)	0.006	0%	3	1.02 (0.80, 1.29)	0.89	76%
	Other (Caucasian, Indian, Korean)	2	0.97 (0.75, 0.83)	0.83	0%	1	1.24 (1.06, 1.46)	0.009	NA
GG *vs.* CC	Overall	4	0.70 (0.55, 0.88)	0.003	0%	4	1.17 (0.78, 1.77)	0.44	76%
	Large size	1	0.73 (0.54, 0.98)	0.04	NA	2	1.64 (1.27, 2.12)	0.0002	0%
	Small size	3	0.65 (0.44, 0.96)	0.03	0%	2	0.81 (0.59, 1.10)	0.18	0%
	Chinese	2	0.68 (0.53, 0.88)	0.003	0%	3	1.05 (0.62, 1.77)	0.87	78%
	Other (Caucasian, Indian, Korean)	2	0.81 (0.43, 1.54)	0.53	0%	1	1.59 (1.13, 2.24)	0.007	NA
GC *vs.* CC	Overall	4	0.80 (0.65, 0.98)	0.03	0%	4	1.08 (0.94, 1.25)	0.27	0%
	Large size	1	0.72 (0.55, 1.39)	0.02	NA	2	1.17 (0.98, 1.40)	0.09	0%
	Small size	3	0.91 (0.66, 1.25)	0.56	0%	2	0.95 (0.75, 1.22)	0.71	10%
	Chinese	2	0.80 (0.64, 1.00)	0.05	39%	3	1.04 (0.86, 1.27)	0.67	20%
	Other (Caucasian, Indian, Korean)	2	0.74 (0.39, 1.43)	0.37	0%	1	1.16 (0.91, 1.49)	0.23	NA
GG/GC *vs.* CC	Overall	4	0.76 (0.63, 0.93)	0.007	0%	4	1.10 (0.90, 1.34)	0.36	54%
	Large size	1	0.72 (0.56, 0.93)	0.01	NA	2	1.26 (1.07, 1.50)	0.007	0%
	Small size	3	0.83 (0.61, 1.11)	0.21	0%	2	0.91 (0.73, 1.14)	0.41	17%
	Chinese	2	0.76 (0.62, 0.94)	0.01	0%	3	1.04 (0.79, 1.35)	0.79	60%
	Other (Caucasian, Indian, Korean)	2	0.78 (0.42, 1.45)	0.43	0%	1	1.26 (1.00, 1.59)	0.05	NA

**Table 3:** Stratified effects of miR-146a (rs2910164) on ischemic stroke risk under dominant genetic model (GG/GC *vs*. CC).

Selected Variables		Number of Studies	OR (95% CI)	*p*-Value	*I*^2^ (%)
TOAST	LAA	3	0.80 (0.51, 1.27)	0.34	71%
	SVD	3	1.29 (0.90, 1.84)	0.17	58%
Gender	Male	2	1.06 (0.82, 1.38)	0.65	0%
	Female	2	1.16 (0.45, 2.97)	0.76	86%
Smoker	Yes	2	1.36 (0.91, 2.03)	0.14	0%
	No	2	1.09 (0.86, 1.38 )	0.86	69%
Hypertension	Yes	2	0.87 (0.42, 1.82)	0.72	78%
	No	2	1.30 (0.97, 1.75)	0.08	0%
Diabetes mellitus	Yes	2	1.76 (0.96, 3.21)	0.07	0%
	No	2	1.25 (0.95, 1.63)	0.11	0%
Hyperlipidemia	Yes	2	1.16 (0.78, 1.71)	0.47	17%
	No	2	1.23 (0.80, 1.88)	0.34	42%

TOAST: Trial of Org 10172 in Acute Stroke Treatment; LAA, Large artery atherosclerosis; SVD, Small vessel Disease.

### 2.3. Sources of Heterogeneity

For IS, the subgroup analysis indicated the sample size was the source of the heterogeneity among studies (*p* = 0.001 for allelic model; *p* = 0.0006 for homozygous model; and *p* = 0.02 for dominant model) (data not shown). For CAD, there are no significant heterogeneity existing among all studies.

### 2.4. Sensitivity Analysis

**Figure 4:**
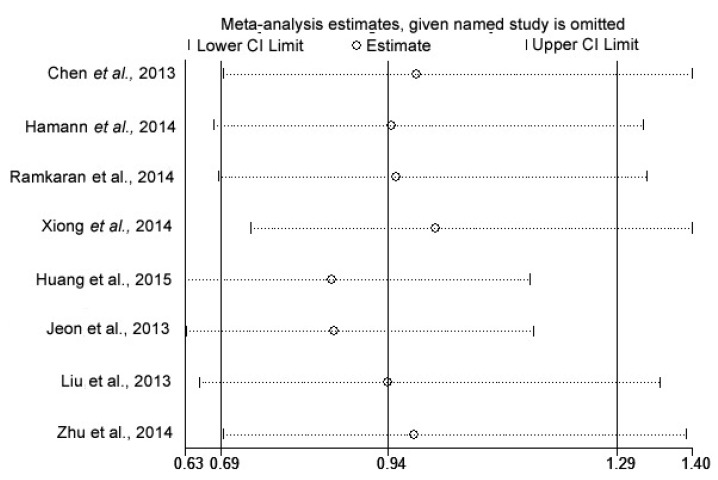
The influence of each study by removal of individual studies in China for G *vs.* C model.

The influence of each study on the pooled ORs and 95% CIs were evaluated by excluding one single study at a time. The corresponding pooled OR was not significantly altered in all genetic models (Figure 4).

### 2.5. Publication Bias

We performed the Begg’s funnel plot and Egger’s test to evaluate the publication bias. The *p* value for Egger’s linear regression tests is shown in Table 4. As the results indicated, no obvious publication bias was observed. And these results were also demonstrated by the shape of funnel plot (not shown).

**Table 4:** Egger’s linear regression test for funnel plot asymmetries.

Group	*p*-Value			
	G *vs.* C	GG *vs.* CC	GC *vs.* CC	GG/GC *vs.* CC
CAD	0.240	0.137	0.965	0.967
IS	0.110	0.191	0.420	0.276

## 3. Discussion

The main findings of our meta-analysis show that rs2910164 is associated with the risk of CAD significantly in allelic model (OR = 0.86), homozygous model (OR = 0.70), heterozygous model (OR = 0.80) and dominant model (OR = 0.76). The subjects carrying GG genotype, GG + GC genotype or G allele are at lower risks of CAD. For the susceptibility of IS, there are no significant associations between rs2910164 and total studies. However, in subgroup analysis by sample size and ethnicity, the GG, GG + GC and G allele of rs2910164 are found to be associated with higher risks of IS in large size group and in Koreans, under homozygous and dominant model.

Although a very recent meta-analysis was performed to reveal the relationship of rs2910164 and cardio-cerebrovascular diseases, He *et al.* [15], they included fewer studies on CAD and IS than those in our meta-analysis. In our meta-analysis, we included two more studies, with 737 cases and 731 controls, for CAD and IS analysis. We further conducted a more comprehensive subgroup analysis for IS by TOAST, gender, smoking, hypertension, diabetes mellitus and hyperlipidemia. Furthermore, we found some surprisingly different results between our meta-analysis and He *et al.*’s in CAD. Although we found a similar lower CAD risks for G allele *vs.* C allele, our meta-analysis also demonstrated a lower risks of CAD for GG/GC and GG genotype *vs.* CC carriers (Figure 2 and Table 2). However, He *et al*.’s meta-analysis stated a higher risk for GG carriers *vs.* CC carriers.

In the sensitivity analysis, no significant changes were found after omitting each study one at a time, indicating the relative stability and credibility of the results of our meta-analysis.

We conducted a subgroup analysis by sample size and ethnicity, and observed that rs2910164 was associated with the risks of CAD and IS in the large sample group. However, only one study was included in the large size subgroup in CAD, and two studies were included in IS. We also found a higher risk of IS in Korean for G allele, GG/GC and GG genotypes of miR-146a, and once again, only one study is included in this subgroup. Thus, further studies are needed to confirm these results.

Since no associations were found between rs2910164 and IS, we performed subgroup analysis by TOAST, gender, smoking, hypertension, diabetes mellitus and hyperlipidemia (Table 3). No significant relations were found in all the analyses. But some studies lack sufficient data for the subgroup analysis. For example, the study of Huang *et al*. [11] lacks TOAST data, while Jeon *et al*. and Zhu *et al*. lack gender, smoking, hypertension, hyperlipidemia and diabetes mellitus data [12,16]. Interestingly, in the study of Zhu *et al*. [16], no significant correlations were found between rs2910164 and overall subjects, though when they divided the patients to LAA and SVD according to TOAST typing, significantly higher frequencies in LAA-caused IS were found in the CC genotype and C allele. However, the results of Zhu *et al*. [16], focusing on LAA-caused IS was contradictory to the study of Jeon *et al*. [12]. This may be the result of different target genes, geographical ethnic groups, sample size, *etc.* Further studies are still needed to resolve this discrepancy.

CAD and IS are both manifestations of atherosclerosis. Most studies included in the present meta-analysis found dyslipidemia in the case group. Previous studies have indicated atherosclerosis as an inflammatory process. Thus, anti-inflammatory treatments decrease the risk of atherosclerosis. miR-146a regulates the NF-κB-induced inflammatory process by targeting interleukin-1 receptor-associated kinase 1 (IRAK-1) and TNF receptor-associated factor 6 (TRAF-6). IRAK-1 and TRAF-6 are upstream regulators of NF-κB activation, and major signal transducers of the Toll-like receptor (TLR) system [9]. The SNP in the pre-miR-146a changed the G to C allele, thus influence the mature miR-146a production, and subsequently influence the inflammatory process of atherosclerosis. Interestingly, the GG, GG + GC genotype and G allele are associated with lower susceptibility of CAD in overall subjects and large sample size group. On the contrary, GG, GG + GC genotype and G allele are related to higher risk of IS in large sample size group. This could be explained by the target gene selection of miR-146a and the pathological differences of these two diseases [10,17,18,19,20]. Furthermore, the endothelial dysfunction induced by the inflammatory process is considered to be the first step of atherosclerosis. Further investigations are needed to reveal whether or not rs2910164 affects endothelial mature miR-146a production and the effects of this variation might have on endothelial functions.

The results of the present meta-analysis should be interpreted carefully because of the following limitations. Firstly, the number of patients was relatively small, and may influence the outcomes. After a very comprehensive literature search from several different databases, only a total of eight studies were included in the present meta-analysis. Among them, four are related to CAD (1265 cases and 1241 controls) and four are related to IS (1873 cases and 1856 controls). The overall OR only indicated the associations between rs2910164 and CAD. However, no associations were found between rs2910164 and IS risk. The second limitation is that the clinicopathological characteristics or disease subtypes are limited in most of these studies. Although we conducted subgroup analysis by clinicopathological characteristics in IS, only half of the included studies provide the necessary data. And for CAD, no such characteristics were available. Thirdly, CAD and IS are both multi-factorial diseases influenced by both genetic and environmental factors. The gene-gene and gene-environment interactions may play important roles in the function of rs2910164, but most studies lack information about environmental exposure and multiple SNPs in miRNA-encoding genes. The fourth limitation lies in the ethnicity of the subjects. Most of the patients were Asians in the present study and this limited the general application of the results to other populations.

In conclusion, the current meta-analysis suggests a decreased risk of CAD for GG, GG + GC genotype and G allele of rs2910164, while rs2910164 is not associated with the risk of IS. Thus rs2910164 might be recommended as a predictor for susceptibility of CAD, but not IS. However, the results of this meta-analysis should be interpreted with caution because of the heterogeneity among study designs. Further study is needed to evaluate the association of rs2910164 and these two diseases, especially in a large sample size, in Caucasians, and with clinicopathological characteristics.

## 4. Methods

### 4.1. Publication Search Strategy and Inclusion Criteria

Published studies were systematically searched by Mei-hua Bao and Yan Xiao; the electronic databases Pubmed, Embase, Cochrane Central Register of Controlled Trials (CENTRAL), Chinese National Knowledge Infrastructure (CNKI) and Chinese Biomedical Literature Database (CBM) were searched for the following terms: “Coronary artery disease (CAD)” or “ischemic stroke (IS)” and (“miR-146a” or “miRNA-146a” or “microRNA-146a”) and (“polymorphism” or “mutation” or “variant” or “SNP” or “single nucleotide polymorphism”), without restriction on language. The deadline for publication was 15 March, 2015. All the results from the databases were screened. First, we screened the title. If the titles fulfilled our criteria, we then screened the abstract. We retrieved the full text if the abstract was interesting. All eligible studies were retrieved manually for other potentially relevant studies from their references. We contacted the authors for related data when they were unavailable in the original publications.

Inclusion Criteria: (a) Case-control design; (b) The association of miR-146a (rs2910164) and CAD or IS risks should be evaluated; (c) The genotype in the control group should be agreed with the Hardy-Weinberg equilibrium (HWE); (d) The data in the publication are sufficient for present estimation. Studies were excluded if any of the following applies: (1) Repeat publications, abstracts, letters or reviews; (2) Studies not meeting all of the inclusion criteria.

### 4.2. Data Extraction

We extracted the information from each eligible publication manually by two investigators independently (Qing-song Zhang and Ji Luo). For each study, the extracted information included: First authors’s name, year of publication, country, ethnicity, genotype method, sex ratio, age, source of controls, diseases, genotype numbers of cases and controls. If we encountered discrepancies during data extraction, it was resolved by a consensus achieved by the third author (Jian-ming Li).

### 4.3. Quality Assessment

The quality of the included studies was evaluated by the following aspects: source of cases, source of controls, specimens used for determining genotypes, total sample size and evidence of HWE. The quality scores ranged from 0–15, higher scores indicating better quality [21]. The quality evaluation was performed by two authors independently (Juan Zhao and Guang-yi Li). Meetings were held to resolve any discrepancies in the assessment process.

### 4.4. Statistical Methods

χ^2^-test was used to evaluate the HWE of the control group polymorphism. If *p* < 0.05, it was considered to be deviated from HWE. To evaluate the association between miR-146a (rs2910164) and disease (CAD and IS) risk, the crude odds ratio (OR) with 95% confidence interval (CI) was used. The pooled ORs were calculated using genetic model of allelic model (G *vs.* C), homozygous (GG *vs.* CC), heterozygous (GC *vs.* CC) and dominant (GG/GC *vs.* CC) model, and the statistical significance was determined by the *Z*-test, and *p* < 0.05 was considered to be statistically significant. Subgroup analysis was conducted according to the sample size. Total samples less than 1000 was treated as small, and otherwise as large.

The statistical heterogeneity between studies was evaluated by *I*-square statistical test, which was not dependent on the number of studies in the meta-analysis [22]. If there was an obvious heterogeneity among the studies (*I*^2^ > 50%), the random-effects model (the DerSimonian and Laird method) was used for the meta-analysis [23]. Otherwise, the fixed-effect model using the Mantel-Haenszel method was used [24]. Sensitivity analysis was performed to assess the effects of individual study on pooled results and the stability of results. The publication bias was detected with Begg’s funnel plot, and Egger’s linear regression method, and *p* < 0.05 was considered to be statistically significant [25]. All statistical analysis was performed using the STATA 12.0 software (StataCorp, College Station, TX, USA) and Revman 5.3.
